# CTH Promotes Liver Cancer Progression and Exploratory Evaluation Supports Enhanced Antitumor Activity of PPG Plus GPC3 CAR‐T Cells

**DOI:** 10.1155/jimr/3351382

**Published:** 2026-10-16

**Authors:** Yuxin Guo, Man Yu, Xilin Zhu, Wei Li, Ying Liu, Xiaopan Wu

**Affiliations:** ^1^ Department of Biochemistry and Molecular Biology Institute of Basic Medical Sciences and School of Basic Medicine Chinese Academy of Medical Sciences and Peking Union Medical College Beijing 100005 China cacms.ac.cn; ^2^ Department of Interventional Radiology The Affiliated Hospital of Qingdao University Qingdao 266003 Shandong China qdu.edu.cn

**Keywords:** autophagy, CD8, CTH, HCC, sulfhydration

## Abstract

Although hydrogen sulfide (H_2_S) is a key regulator in cancer biology, the mechanism by which the H_2_S‐producing enzyme cystathionine‐γ‐lyase (CTH) functions in hepatocellular carcinoma (HCC) remains elusive. Using the slow‐releasing H_2_S donor GYY4137, we observed enhanced migration and invasion in HuH7 and HepG2 cells. Subsequently, we observed consistent protumorigenic effects of CTH not only in vitro but also in subcutaneous and orthotopic mouse models. Here, our findings suggest that CTH‐H_2_S sulfhydrates Rab7a, which may be associated with HCC progression. We further showed that CTH modulates the expression of autophagy‐related proteins in liver tumor cells. In nude mouse subcutaneous xenograft experiments, compared with the vehicle group, the combination of DL‐propargylglycine (PPG, CTH inhibitor) and GPC3 CAR‐T exhibited better antitumor efficacy compared to PPG or CAR‐T alone. Our findings suggest that CTH modulation was associated with altered Rab7a sulfhydration and autophagy and that combined PPG and GPC3 CAR‐T therapy exerts better antitumor efficacy.

## 1. Introduction

Hydrogen sulfide (H_2_S) mediates protein sulfhydration, modifying reactive cysteine thiol groups to form sulfhydrated groups [1]. This modification enhances the reactivity of cysteine residues, often leading to conformational changes in proteins, thereby modulating their enzymatic activity or function and acting as an important switch or regulatory factor [1]. Early proteomic studies suggested that protein sulfhydration may affect a substantial fraction of hepatic proteins [2]. A study reveals that protein sulfhydration is crucial in liver injury [3]. Enzymes such as cystathionine‐β‐synthase (CBS), cystathionine‐γ‐lyase (CSE/CTH), and cysteine aminotransferase (CAT)‐3‐mercaptopyruvate sulfurtransferase (MPST) metabolize methionine and cysteine to generate H_2_S[2, 4]. S‐sulfhydration plays a crucial role in coordinating a variety of physiological processes, including inflammation, endoplasmic reticulum stress, and signal transduction. Emerging evidence demonstrates that H_2_S‐mediated S‐sulfhydration of key proteins can modulate autophagy in cancer [5].

The occurrence of cancer in the body is closely related to the dysfunction of immune cells [6]. In recent years, adoptive cell therapy has emerged as a successful immunotherapy strategy for cancer [7, 8]. Cellular immunotherapy possesses the ability to sense diseases and mount dynamic responses [9]. Current research on hepatocellular carcinoma (HCC) has seen rapid advancements in the study of engineered immune cells targeting liver tumors, such as GPC3 CAR T cells [10], which have shown promising therapeutic potential when combined with small‐molecule agents in the treatment of HCC [11].

In this study, we demonstrated that CTH promotes liver cancer progression. Furthermore, CTH‐associated H_2_S signaling is linked to increased Rab7a sulfhydration, and our data suggest that it modulates autophagy. In addition, high CTH expression was associated with a larger tumor diameter and poorer overall survival in a single‐center HCC cohort. Therapeutically, we showed that targeting CTH with its pharmacological inhibitor DL‐propargylglycine (PPG), in combination with CAR‐T therapy, showed greater antitumor activity than either monotherapy in a xenograft model.

## 2. Materials and Methods

### 2.1. Cell Lines and Culture Conditions

The human hepatoblastoma‐derived cell line HepG2 (RRID: CVCL_0027) and the HCC cell line HuH7 (RRID: CVCL_0336) were used in this study. The HepG2 cell line was originally established from the liver tissue of a 15‐year‐old male Caucasian patient with hepatoblastoma, whereas the HuH7 cell line was originally established from the liver tissue of a 57‐year‐old male Japanese patient with HCC.

Both cell lines were obtained from the National Infrastructure of Cell Line Resource (Beijing, China) in 2020. Cell line authentication was performed by the supplier using short tandem repeat profiling, which showed 100% concordance with the corresponding reference profiles. According to the International Cell Line Authentication Committee database, neither HepG2 nor HuH7 is listed as a commonly misidentified or cross‐contaminated cell line. Cells were routinely tested for mycoplasma contamination using a detection kit and remained negative throughout the study.

HepG2 and HuH7 cells were cultured in Dulbecco’s modified Eagle medium supplemented with 10% fetal bovine serum and 1% penicillin–streptomycin. Cells were maintained at 37°C in a humidified atmosphere containing 5% CO_2_.

### 2.2. Stable CTH‐Overexpressing and CTH‐Knockdown Cell Lines

To generate stable CTH‐overexpressing and CTH‐knockdown HuH7 cell lines, lentiviral particles expressing CTH, CTH‐specific short hairpin RNA (sh‐CTH), or the corresponding controls (empty vector [Vec] and nontargeting shRNA [Nc]) were produced in HEK293T cells by cotransfection of the corresponding transfer plasmids with lentiviral packaging plasmids maintained in our laboratory.

HuH7 cells were transduced with the corresponding lentiviral particles at a multiplicity of infection (MOI) of 5 in serum‐free DMEM. After ~6 h, the medium was replaced with a complete medium, and cells were cultured for an additional 72–96 h. Stable cell lines were subsequently established by selection with 2 μg/mL puromycin.

### 2.3. Animal Experiments

All animal experiments were approved by the Ethics Committee (Approval Number ACUC‐XMSB‐2025‐002). Four‐week‐old male BALB/c nude mice and 6‐week‐old male NOG mice were purchased from the Center of Medical Experimental Animals of the Chinese Academy of Medical Sciences (Beijing, China).

#### 2.3.1. Subcutaneous Xenograft Model

A total of 5 × 10^6^ stable sh‐CTH or Nc‐expression HuH7 cells suspended in 200 μL of phosphate‐buffered saline (PBS) were injected subcutaneously into the axillary region of nude mice to evaluate the effect of CTH knockdown on tumor‐forming capacity. All mice were euthanized on day 28 for the final analysis.

#### 2.3.2. Orthotopic Tumorigenesis Model

Stable CTH‐ or Vec‐expression HuH7 cells were inoculated into the liver through orthotopic intrahepatic injection to evaluate tumor‐forming capability within the hepatic microenvironment. Liver tumor formation was monitored over a period of 4 weeks.

### 2.4. Plasmid Construction and Transfection

The CTH and CTH mutant (CTH mut) plasmids were constructed using the 3 × FLAG‐CMV−14 vector. RAB7a and RAB7 mutant (CCC) plasmids were constructed using the LV‐ef1a‐cycm‐IRES‐GFP vector. Plasmid construction services were provided by Shanghai GeneRay Biotech Co., Ltd. Cells were transfected with the indicated plasmids using Lipofectamine 3000 reagent (Thermo Fisher Scientific, USA) and cultured for 48 h before subsequent experiments.

### 2.5. Transwell Migration and Invasion Assay

Cell migration and invasion were evaluated using transwell chambers (Corning, 3422). For migration assays, 2 × 10^5^ cells suspended in serum‐free medium were seeded into the upper chamber, while the lower chamber was filled with 600 μL of medium containing 20% FBS. After incubation for 48 h, nonmigrated cells on the upper surface of the membrane were removed. The migrated cells were fixed with 4% neutral formaldehyde, stained with 0.1% crystal violet, and imaged under a microscope.

For invasion assays, the upper chamber was precoated with matrigel diluted 1:3 in a serum‐free medium before cell seeding. The subsequent procedures were performed following the same protocol as the migration assay.

### 2.6. Colony Formation Assay

Cells were transfected with the indicated plasmids and cultured for 48 h. Cells were then reseeded in 12‐well plates at a density of 2000 cells per well. After culture for 14 days, with medium replacement every 5 days, colonies were fixed with 4% neutral formaldehyde, stained with 0.1% crystal violet, and photographed.

### 2.7. Measurement of Hydrogen Sulfide (H_2_S)

HuH7 cells were transfected with Vector, CBS, CTH, and MPST plasmids. At 48 h after transfection, the intracellular H_2_S level was measured using an H_2_S Detection Kit (Geruisi Biotechnology, G0133W).

Cells were washed with PBS, and 1 mL of extraction buffer was added per 5 × 10^6^ cells. Cells were lysed by ultrasonication on ice at 20% power using 3 s pulses with 10 s intervals for a total of 30 cycles. Lysates were centrifuged at 12,000 rpm for 10 min at 4°C, and the supernatants were collected for analysis. Cell lysates and detection reagents were added to the measurement wells, whereas ddH_2_O was added to the blank wells. After incubation for 15 min at room temperature, the absorbance was measured at 665 nm. The H_2_S release was calculated according to the manufacturer’s instructions.

### 2.8. Western Blot (WB) Analysis

Polyvinylidene fluoride membranes containing the target proteins were incubated overnight at 4°C with primary antibodies (Table S1). After washing with tris‐buffered saline containing Tween 20, membranes were incubated with secondary antibodies at room temperature for 2 h. Following additional washing, protein signals were detected by chemiluminescence.

### 2.9. Biotin Switch Assay for Protein Sulfhydration

Total cellular proteins were extracted using a protein lysis buffer. Protein samples were incubated with blocking buffer at 50°C for 20 min and subsequently precipitated with acetone at −20°C. The proteins were then labeled with 4 mM Biotin‐HPDP under light‐protected conditions for 75–90 min. Biotin‐labeled proteins were incubated with streptavidin beads overnight at 4°C. After washing with HENS buffer, the bound proteins were eluted using loading buffer. Protein sulfhydration levels were subsequently detected by WB analysis.

### 2.10. mRFP‐GFP‐LC3 Adenoviral Infection

Cells were seeded on coverslips in 24‐well plates. After 24 h, cells were infected with mRFP‐GFP‐LC3 adenovirus for 2 h. The medium was then replaced, followed by the indicated treatments, including plasmid transfection or drug administration. After an additional 48 h of culture, the cells were collected and examined.

### 2.11. Clinical Specimens and Immunohistochemistry

A total of 122 HCC tissue samples were collected from patients who underwent liver resection at the Affiliated Hospital of Qingdao University (Qingdao, China) between 2010 and 2016. Immunohistochemical staining for CTH was performed by Wuhan Servicebio Technology Co., Ltd. CTH staining was independently evaluated by two investigators. Tumor samples with >50% positively stained cells were classified as high CTH expression, while those with ≤50% positively stained cells were classified as low CTH expression. Kaplan–Meier curves were generated for survival analysis. The use of human samples in this study was specifically reviewed and approved by the Institutional Review Board of the Affiliated Hospital of Qingdao University and the Institute of Basic Medical Sciences, Chinese Academy of Medical Sciences (Approval Number 012‐2011).

### 2.12. Generation of GPC3 CAR‐T Cells

The GPC3 CAR vector was provided by Yunzhou Biotechnology. Peripheral blood mononuclear cells (PBMCs) were isolated from healthy donors using a human PBMC separation solution (TBD, LTS1077). T cells were stimulated with anti‐CD3 and anti‐CD28 antibody (2 μg/mL each) for 48 h and expanded in RPMI 1640 medium supplemented with 100 U/mL interleukin‐2 (IL‐2), 10% FBS, and 50 μM β‐mercaptoethanol. Activated T cells (5 × 10^5^) were transduced with GPC3 CAR lentivirus or a control lentivirus lacking the CAR gene at an MOI of 10. Lentiviral transduction was performed by centrifugation at 37°C and 2000 *× g* for 99 min. Following centrifugation, cells were incubated for 6–8 h, after which the medium was replaced with a complete medium containing IL‐2. Transduced T cells were expanded in an IL‐2‐containing medium at a density of 0.5–1 × 10^6^ cells/mL.

### 2.13. In Vitro Cytotoxicity Assay

The cytotoxic activity of CAR‐T cells against tumor cells was assessed using the CytoTox 96 Nonradioactive Cytotoxicity Assay (Promega, G1780). A preliminary experiment was performed to determine the optimal target cell density. Target tumor cells were seeded into 96‐well plates at 5 × 10^3^ cells per well. CAR‐T cells were added at effector‐to‐target ratios of 5:1, 2.5:1, and 1.25:1. In PPG‐treatment groups, 200 μM PPG (Solarbio, D6010) was added to the designated wells for 24 h. After treatment, 50 µL of the supernatant was collected from each well and mixed with the CytoTox reagent. The reaction mixture was incubated in the dark for 30 min and terminated by adding the stop solution. Absorbance was measured at 490 nm within 1 h. Specific cytotoxicity was calculated using the following formula:

Cytotoxicity%=experimental release− effector spontaneous release− target spontaneous release/target maximum release− target spontaneous release×100%.

### 2.14. Flow Cytometry Analysis

Mononuclear cells were stained using flow cytometry fixation and permeabilization buffers (Thermo Fisher Scientific, 00‐5523). For intracellular staining, cells were treated with a cell stimulation cocktail (Thermo Fisher Scientific, 00‐4975‐93) for 4 h before staining. Antibody information for flow cytometry is provided in Table S2.

The fluorochrome panels included CD45‐PerCP/Cyanine5.5, CD3‐FITC, CD8‐APC, CD223 (LAG‐3)‐PE/Cyanine7, PD‐1‐Brilliant Violet 421, TNF‐α‐Brilliant Violet 421, and Granzyme B (GZMB)‐PE. Samples were acquired using a CytoFLEX S (Beckman Coulter) flow cytometer. The voltage settings for each channel were as follows: FSC: 119, SSC: 86, PerCP‐Cy5.5: 604, FITC: 171, APC: 517, PE: 162, PC7: 617, and PB450 (BV421): 28. Compensation was performed using single‐stained antibody capture compensation beads to correct for the spectral overlap. The compensation parameters are provided in Tables S3 and S4. Data were analyzed using FlowJo software.

### 2.15. Combined Antitumor Efficacy of PPG and GPC3 CAR‐T Cells

The combined antitumor efficacy of PPG and GPC3 CAR‐T cells was evaluated in subcutaneous xenograft HCC models. Mice were inoculated subcutaneously with 5 × 10^6^ HuH7 cells. Ten days after inoculation, mice were randomly assigned to different groups. Mice in the corresponding groups received either a single intravenous injection of 1 × 10^7^ GPC3 CAR‐T (CAR‐T) or mock‐T cells, together with daily intraperitoneal administration of PPG (50 mg/kg) or vehicle control (ddH_2_O). All mice were euthanized on day 28 for the final analysis.

### 2.16. Exploratory Bliss Independence Analysis

Under the Bliss independence framework, the expected effect of a combination is derived from the effects of the individual treatments under the assumption of independent action [12]. Based on this model, the interaction between PPG and GPC3 CAR‐T cells was assessed exploratorily using mean endpoint tumor volumes on day 28.

The tumor‐growth inhibition rate (IR) for each treatment group, relative to the vehicle group, was calculated as follows:

IR=1−mean tumor volume of treatment group/mean tumor volume of vehicle group.

The expected IR for the combination under the Bliss independence model was calculated as follows:

IRexpected= IRPPG+ IRCAR-T−IRPPG× IRCAR-T.

The observed‐to‐expected residual tumor volume ratio was calculated as follows:

1− IRcombination/1− IRexpected.

A ratio of <1, = 1, or >1 indicates that the observed residual tumor burden was lower than, consistent with, or higher than that predicted under the Bliss independence model, respectively. Given that this analysis was based on a single dose combination and endpoint tumor volume data, it was interpreted descriptively and was not considered, by itself, to establish pharmacological synergy.

### 2.17. Statistics

Statistical analyses and graphical presentation were performed using GraphPad Prism version 9.0 (GraphPad Software, La Jolla, CA, USA). Data are presented as the mean ± standard deviation (SD). All experiments were performed with at least three independent biological replicates (*n* ≥ 3). Comparisons between the two groups were performed using an unpaired two‐tailed Student’s *t*‐test. For comparisons among multiple groups, one‐way analysis of variance (ANOVA) followed by appropriate post hoc multiple‐comparison tests was used. The specific statistical tests applied for each experiment are indicated in the corresponding figure legends. A two‐sided *p* value < 0.05 was considered statistically significant.

## 3. Results

### 3.1. H_2_S Promotes Migration and Invasion in Liver Cancer Cells

H_2_S has emerged as a key gaseous signaling molecule involved in various physiological and pathological processes, including the regulation of cancer cell behavior[13]. In this study, we employed GYY4137 (morpholin‐4‐ium 4‐methoxyphenyl(morpholino)phosphinodithioate), a slow‐releasing H_2_S donor, to treat two liver cancer cells, HuH7 and HepG2. In HepG2 cells, GYY4137 significantly promoted cell migration and invasion (Figure 1A–D). A similar promotive effect was observed in HuH7 cells, indicating that the compound consistently enhances these malignant behaviors in both cell lines (Figure 1E–H). Given the critical role of endogenous H_2_S production, we focused on three major enzymatic sources: CBS, CTH, and MPST. To investigate their individual contributions, we constructed expression plasmids for each of these enzymes. Following 48 h of overexpression in HuH7 cells, migration assays revealed that CTH specifically and significantly promoted cell migration, suggesting a distinct role for CTH‐derived H_2_S in enhancing metastatic potential (Figure 1I,J). We next performed experiments to measure H_2_S production. HuH7 cells overexpressing CBS, CTH, or MPST were harvested and lysed by sonication, and the supernatants were used to determine H_2_S levels. Among the three groups, CTH overexpression led to the highest H_2_S production (Figure 1K). We speculate that the enhanced cell migration observed specifically in the CTH‐overexpressing group may be attributed to the greater H_2_S‐producing capacity of CTH in HuH7 cells, whereas the H_2_S generated by CBS and MPST is insufficient to significantly affect cell migration.

**Figure 1:**
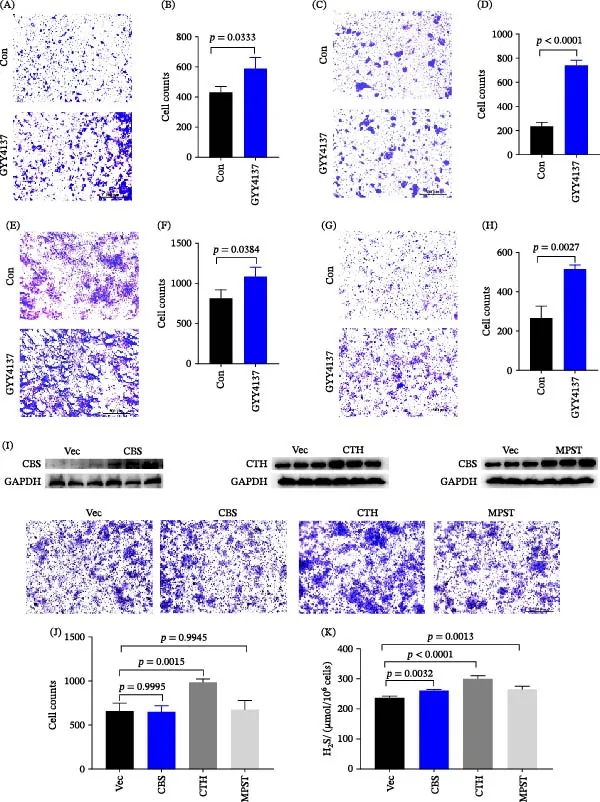
H_2_S promotes migration and invasion of liver cancer cells. (A–H) A slow‐releasing H_2_S donor, GYY4137 (morpholin‐4‐ium 4‐methoxyphenyl (morpholino) phosphinodithioate), treatment (200 μM, 24 h) affected migration and invasion in HepG2 cells (A–D) and HuH7 cells (E–H), with migration shown in (A, B) and (E, F) and invasion shown in (C, D) and (G, H), respectively. Representative transwell images are shown in (A), (C), (E), and (G), and the corresponding quantifications are shown in (B), (D), (F), and (H). Migrated or invaded cells were quantified as cell number per field using ImageJ. Migration and invasion data were obtained from three independent biological experiments (*n* = 3). GYY4137 treated cells were compared with controls (Con, equal volume of ddH_2_O) using an unpaired two‐tailed Student’s *t*‐test. Scale bars, 500 μm. (I, J) Cystathionine‐γ‐lyase (CTH), cystathionine‐β‐synthase (CBS), or 3‐mercaptopyruvate sulfurtransferase (MPST) overexpression in HuH7 cells were validated by western blot (WB) (I). Representative transwell migration images (I) and quantification of migrated HuH7 cells (J) show the effects of overexpressing CTH, CBS, or MPST. Data of WB and migration were obtained from three independent biological experiments (*n* = 3). (K) H_2_S production in HuH7 cells was assessed following overexpression of CTH, CBS, or MPST. Data were obtained from three independent biological experiments (*n* = 3), with four technical replicates per condition in each experiment. For subfigures (J) and (K), one‐way ANOVA followed by Dunnett’s multiple‐comparisons test was used, with the empty‐vector (Vec) group as the control. All data are presented as mean ± SD; error bars represent SD. *p* values are shown in the figure; comparisons with *p*  < 0.0001 are indicated as *p*  < 0.0001.

### 3.2. CTH Promotes Malignant Phenotypes and Tumor Growth in Liver Tumor Models

After confirming the promotive function of CTH‐derived H_2_S on the migration and invasion of liver tumor cells, we first performed CTH overexpression, knockdown, and CTH inhibitor PPG treatment on HuH7 cells, respectively, followed by further validation of their functional effects. We validated the efficiency of CTH overexpression and knockdown in HuH7 cells, HepG2 cells, and stably transfected HuH7 cell lines (Figure S1A–C). The cell migration assay showed that the CTH knockdown group exhibited opposite outcomes to the CTH overexpression group: the migration ability of HuH7 cells in the sh‐CTH group was significantly reduced (Figure 2A,B). In addition, the migration ability of HuH7 cells in the PPG‐treatment group was significantly decreased compared to the control group (Figure 2C). We also validated these findings through cell invasion assays: the CTH overexpression group showed enhanced invasion ability in HuH7 cells, while the sh‐CTH group exhibited significantly reduced invasion ability, and the PPG‐treatment group showed weakened invasion capability (Figure 2D–F). Consistent results were observed in HepG2 cells: the CTH overexpression group displayed enhanced migration and invasion abilities, the sh‐CTH group showed significantly reduced migration and invasion, and the PPG‐treatment group exhibited decreased migration and invasion abilities (Figure S1D–I).

**Figure 2:**
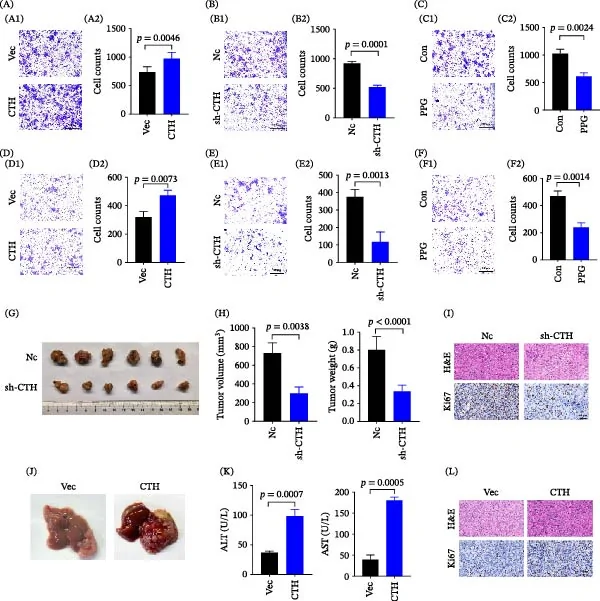
CTH promotes liver tumor growth and progression‐associated phenotypes. (A–F) Representative images (A1–F1) and corresponding quantification (A2–F2) from transwell assays show HuH7 cell migration (A–C) and invasion (D–F) following CTH overexpression (A, D), CTH knockdown (B, E), or CTH inhibitor (DL‐propargylglycine, PPG) treatment (200 μM, 24 h; C, F). Migrated or invaded cells were quantified as cell number per field using ImageJ. Data were obtained from three independent biological experiments (*n* = 3). The indicated experimental group was compared with the corresponding control group (Vec for CTH overexpression, Nc for CTH knockdown, and untreated control for PPG treatment) using an unpaired two‐tailed Student’s *t*‐test. Scale bars, 500 μm. (G–I) Nude mice were subcutaneously injected with HuH7 cells stably expressing sh‐CTH or negative‐control shRNA (Nc) (*n* = 6 mice per group). (G) Image shows excised xenograft tumors. (H) Tumor volume and tumor weight were measured at the experimental endpoint and compared between the sh‐CTH and Nc groups using an unpaired two‐tailed Student’s *t*‐test. (I) Representative hematoxylin and eosin (H&E)‐ and Ki67‐stained tumor sections. Scale bars, 50 μm. (J–L) Immunodeficient mice were injected with HuH7 cells stably overexpressing CTH or the empty‐vector control (Vec) (*n* = 3 mice per group). (J) Representative images show orthotopic liver tumors. (K) Serum alanine aminotransferase (ALT) and aspartate aminotransferase (AST) levels were measured and compared between the CTH‐overexpression and Vec groups using an unpaired two‐tailed Student’s *t*‐test. (L) Representative H&E‐ and Ki67‐stained tumor sections. Scale bars, 50 μm. All data are presented as mean ± SD; error bars represent SD. *p* values are shown in the figure.

To evaluate the role of CTH in liver tumor development, we employed two animal models: subcutaneous and orthotopic tumor implantation. Subcutaneous injection of Nc control or sh‐CTH‐stable HuH7 cells into nude mice resulted in attenuated tumor formation in the sh‐CTH group (Figure 2G,H). In the sh‐CTH group, H&E staining of tumor sections revealed lower cellularity and a looser tissue architecture, while Ki67 staining showed fewer Ki67‐positive cells compared with the Nc control group (Figure 2I). Orthotopic implantation of Vec control or CTH‐overexpressing stable HuH7 cells into the livers of NOG immunodeficient mice revealed enhanced tumorigenicity in the CTH‐overexpressing group (Figure 2J). Additionally, mice in the CTH‐overexpressing group exhibited increased serum levels of ALT and AST, suggesting liver damage (Figure 2K). In the CTH group, H&E staining of liver tumor sections revealed higher cellularity and a more compact tissue architecture, while Ki67 staining showed more Ki67‐positive cells compared with the Vec control group (Figure 2L). Collectively, these results indicate that CTH promotes malignant phenotypes in liver tumor cells and tumor growth in the xenograft and orthotopic models examined.

### 3.3. CTH Modulation Is Associated With Altered Rab7a Sulfhydration

Our lab has previously demonstrated that ATF6 promotes Rab7a sulfhydration and positively regulates CTH expression [14, 15]. Existing studies have confirmed that H_2_S serves as a key mediator of protein sulfhydration [16]. Given that CTH is a critical enzyme responsible for H_2_S production, we hypothesize that CTH‐dependent H_2_S production may contribute to Rab7a sulfhydration. Consistent with this possibility, the biotin switch assay showed that CTH overexpression significantly increased Rab7a sulfhydration, whereas shRNA‐mediated CTH knockdown reduced the Rab7a sulfhydration signal (Figure 3A,B). To assess whether H_2_S availability contributes to the effect of CTH on Rab7a sulfhydration, HepG2 cells were treated with the H_2_S donor GYY4137. Cells were divided into four groups: Vec, CTH, Vec + GYY4137, and CTH + GYY4137. Rab7a input levels were comparable among all groups. Compared with the Vec control, Rab7a sulfhydration levels were increased in the CTH, Vec + GYY4137, and CTH + GYY4137 groups (Figure S2A). No significant difference was observed between the Vec + GYY4137 and CTH + GYY4137 groups, and both groups showed higher Rab7a sulfhydration than that of the CTH group (Figure S2A). This interpretation was further supported by CTH knockdown experiments. HepG2 cells were transfected with sh‐CTH or Nc plasmids and subsequently treated with 200 μM GYY4137 for 24 h. Compared with the Nc group, Rab7a sulfhydration was decreased in the sh‐CTH group, whereas treatment with GYY4137 significantly increased Rab7a sulfhydration in Nc‐transfected cells (Figure S2B). Importantly, GYY4137 treatment restored Rab7a sulfhydration in sh‐CTH‐transfected cells compared with the sh‐CTH group (Figure S2B). Collectively, these findings indicate that CTH overexpression and exogenous H_2_S donor treatment are associated with increased detected Rab7a sulfhydration, whereas CTH knockdown reduces this signal. The restoration of Rab7a sulfhydration by GYY4137 in CTH‐knockdown cells is consistent with the contribution of H_2_S availability to this process.

**Figure 3:**
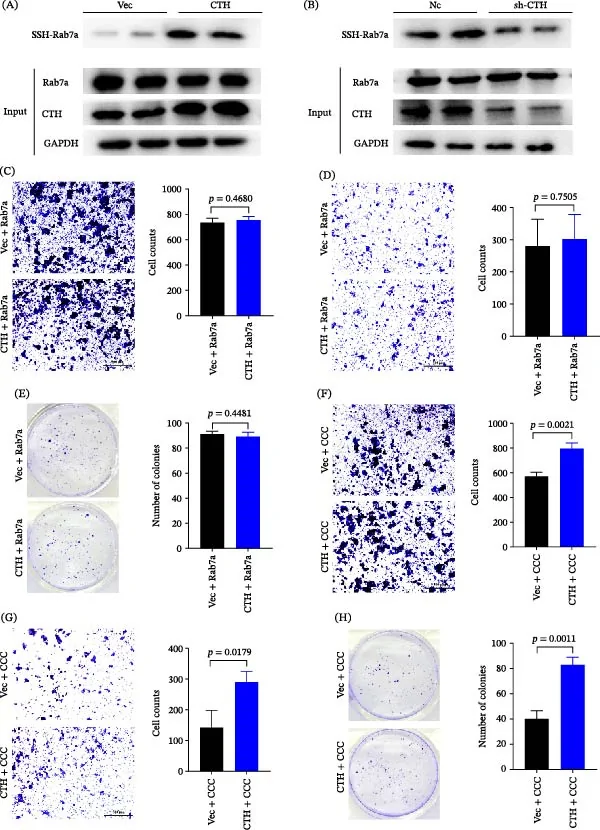
CTH‐H_2_S modulates Rab7a sulfhydration in HepG2 cells. (A, B) WB was used to assess Rab7a sulfhydration levels in HepG2 cells following CTH overexpression (A) or CTH knockdown (B). Data were obtained from three independent biological experiments (*n* = 3). The CTH‐overexpression group in (A) and the CTH‐knockdown group in (B) were compared with the corresponding Vec and Nc controls, respectively. (C–E) HepG2 cells were cotransfected with Vec + Rab7a or CTH + Rab7a plasmids for 48 h, followed by assessment of cell migration (C), invasion (D), and colony formation (E). Data were obtained from three independent biological experiments (*n* = 3). The CTH + Rab7a group was compared with the Vec + Rab7a group using an unpaired two‐tailed Student’s *t*‐test. Data are presented as mean ± SD; error bars represent SD. *p* values are shown in the figure. Scale bars, 500 μm. (F–H) HepG2 cells were cotransfected with Vec + CCC (Rab7a C83/84/143S mutant) or CTH + CCC plasmids for 48 h, followed by assessment of cell migration (F), invasion (G), and colony formation (H). Data were obtained from three independent biological experiments (*n* = 3). The CTH + CCC group was compared with the Vec + CCC group using an unpaired two‐tailed Student’s *t*‐test. Data are presented as mean ± SD; error bars represent SD. *p* values are shown in the figure. Scale bars, 500 μm.

In a previous study, our laboratory generated a Rab7a triple mutant (CCC), in which Cys83, Cys84, and Cys143 were simultaneously replaced with serine. In HepG2.2.15 cells, CCC exhibited reduced Rab7a sulfhydration levels compared with those of wild‐type Rab7a [17]. In the present study, we further validated this finding in HepG2 cells using the previously generated CCC plasmid. Compared with wild‐type Rab7a, CCC showed a significantly reduced Rab7a sulfhydration signal (Figure S2C), suggesting that simultaneous substitution of Cys83, Cys84, and Cys143 reduces the detectable sulfhydration level of Rab7a. We next examined the phenotypic consequences of CCC expression in HepG2 cells. Wild‐type Rab7a overexpression enhanced cell migration and invasion, whereas CCC overexpression did not produce comparable effects (Figure S2D–G). The results from cell migration, cell invasion, and colony formation assays showed that there were no significant differences between the Vec + Rab7a group and the CTH + Rab7a group (Figure 3C–E). In contrast, compared with the Vec + CCC group, the CTH + CCC group significantly enhanced the migration, invasion, and colony formation abilities of HepG2 cells (Figure 3F–H). Collectively, the CTH gain‐ and loss‐of‐function experiments, together with the GYY4137 rescue data, provide pharmacological evidence consistent with CTH‐H_2_S promoting Rab7a sulfhydration.

### 3.4. CTH Is Associated With Altered Autophagy‐Related Processes in Liver Cancer Cells

Autophagy is a conserved process responsible for the degradation of damaged organelles within cells [18]. Dysregulated autophagy has been implicated in HCC development, progression, and therapeutic resistance [19]. Previous studies have demonstrated the role and mechanisms of H_2_S in regulating autophagy in the context of liver diseases [20]. To investigate the contribution of CTH enzymatic activity to autophagy‐related processes in liver cancer cells, we generated a CTH mutant carrying D187A and T189A substitutions, which have been reported to reduce the H_2_S‐producing capacity of CTH [21]. We first examined autophagy‐associated proteins in HuH7 and HepG2 cells following CTH modulation. Overexpression of wild‐type CTH was associated with increased p62 and reduced LC3‐II protein levels, whereas expression of CTH mut did not produce significant changes in these markers (Figure 4A,B). Conversely, CTH knockdown or pharmacological CTH inhibition by PPG resulted in opposite changes in p62 and LC3‐II levels (Figure 4C,D). Collectively, these findings indicate that CTH modulates the steady‐state levels of autophagy‐associated proteins.

**Figure 4:**
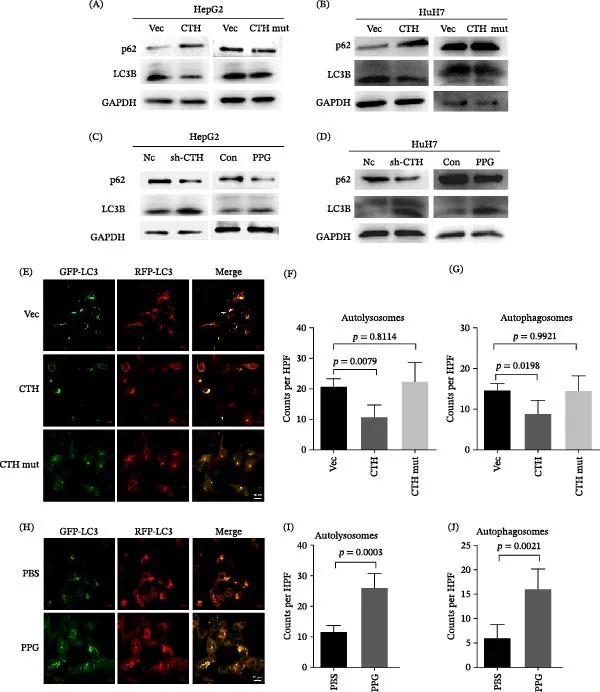
CTH is associated with altered autophagy‐related processes in liver cancer cells. (A–D) Representative WB images show the effects of CTH or CTH mut expression on the autophagy associated markers LC3B and p62 in HepG2 (A) and HuH7 (B) cells, and the effects of CTH knockdown and PPG treatment (200 μM, 24 h) on LC3B and p62 in HepG2 (C) and HuH7 (D) cells. WB was performed in three independent biological experiments (*n* = 3), and representative blots are shown. (E–J) HepG2 cells were transfected with CTH or CTH mut and subsequently analyzed using the mRFP‐GFP‐LC3 reporter assay. Representative images of mRFP‐GFP‐LC3 puncta are shown in (E), with quantification of autolysosomes and autophagosomes shown in (F) and (G), respectively. In parallel, PPG‐treated HepG2 cells were analyzed using the mRFP‐GFP‐LC3 reporter assay. Representative images are shown in (H), with the corresponding quantification of autolysosomes and autophagosomes shown in (I) and (J), respectively. Data were obtained from three independent biological experiments (*n* = 3). For subfigures (F) and (G), statistical significance was analyzed using one‐way ANOVA followed by Dunnett’s post hoc test, with each treatment group compared with the Vec control group. For subfigures (I) and (J), statistical significance was analyzed using an unpaired two‐tailed Student’s *t*‐test, comparing the PPG‐treated group with the vehicle‐treated control group (equal volume of ddH_2_O). Data are presented as mean ± SD; error bars represent SD. *p* values are shown in the figure. Scale bars, 20 μm.

To further evaluate the effects of CTH on LC3‐positive autophagic compartments, we performed an adenoviral mRFP‐GFP‐LC3 reporter assay. In this system, GFP^+^RFP^+^ (yellow) puncta represent autophagosomes, whereas RFP‐only puncta represent acidic autolysosomal compartments in which GFP fluorescence is quenched. CTH overexpression reduced the numbers of both yellow and red puncta (Figure 4E–G), whereas PPG treatment increased both types of puncta (Figure 4H–J). These results show that CTH affects the abundance of LC3 autophagic compartments.

To further investigate the regulation of LC3‐II levels under conditions of inhibited lysosomal degradation, HepG2 and HuH7 cells were treated with bafilomycin A1 (BafA1), a specific inhibitor of vacuolar H^+^‐ATPase that inhibits autolysosomal degradation. Compared with untreated Vec cells, BafA1 treatment resulted in increased LC3‐II protein levels in Vec + BafA1 cells (Figure S3A,B). In contrast, under BafA1 treatment conditions, CTH + BafA1 cells exhibited lower LC3‐II protein levels compared with those of Vec + BafA1 cells (Figure S3A,B). These observations indicate that CTH overexpression is associated with altered LC3‐II levels under conditions of lysosomal inhibition.

### 3.5. Correlation of CTH Expression With Clinicopathological Features and Overall Survival in HCC

Immunohistochemical staining revealed a higher CTH expression in HCC tissues compared with adjacent nontumor tissues (Figure 5A). Kaplan–Meier survival analysis in our cohort of 122 patients indicated that high CTH expression was associated with poorer overall survival (Figure 5B). Furthermore, analysis of clinicopathological features showed that elevated CTH expression was significantly associated with larger tumor diameter (*p* = 0.042) (Figure 5C).

**Figure 5:**
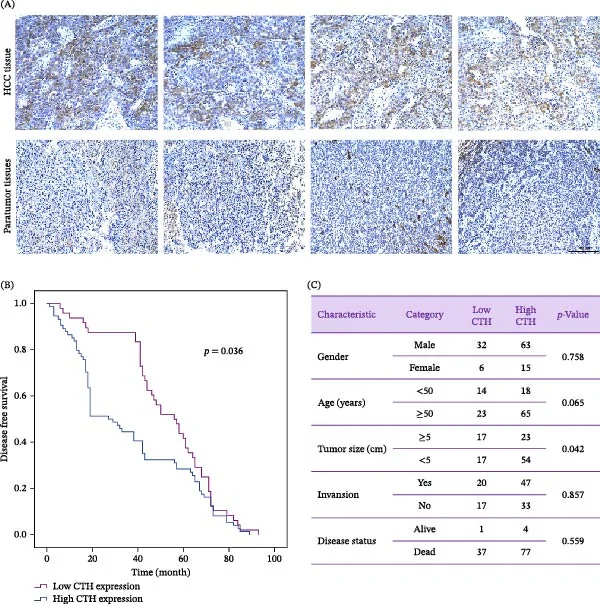
Association of CTH expression with tumor diameter and overall survival in HCC. (A) Representative immunohistochemical images show CTH expression in HCC tissues and adjacent nontumorous tissues. Scale bars, 200 μm. (B) Kaplan–Meier survival curves show the overall survival of 122 patients (*n* = 122) with HCC according to high or low CTH expression. Survival differences between the two groups were evaluated using the log‐rank test (*p* = 0.036). (C) The unadjusted association between CTH expression and clinicopathological variables was evaluated using the *χ* ^2^ test. High CTH expression was significantly associated with larger tumor diameter (*p* = 0.042).

Collectively, these univariate analyses demonstrate an association between high CTH expression, a larger tumor diameter, and poorer overall survival in this cohort. However, these findings do not establish that CTH is independently associated with tumor aggressiveness or patient outcome.

### 3.6. Evaluation of PPG Combined With GPC3 CAR‐T Cells for Antitumor Activity in HCC Models

Our previous studies demonstrated that CTH promotes liver tumor growth. Therefore, we investigated the effects of PPG in combination with GPC3 CAR‐T cells. To evaluate the effect of PPG on CAR‐T cell function, we constructed a GPC3‐targeting CAR‐T vector (Figure 6A), which specifically recognizes GPC3‐expressing liver cancer cells. The CAR‐T lentivirus was packaged and used to transduce human T cells (Figure 6B). CAR‐T cells were expanded and treated with PPG. CFSE dilution analysis showed that PPG increased CAR‐T cell proliferation (Figure 6C). In addition, the PPG + CAR‐T group showed a higher killing rate against HuH7 cells in vitro than that of the CAR‐T group (Figure 6D).

Figure 6Effects of PPG in combination with GPC3 CAR‐T cells in HCC models. (A) Schematic diagram shows the GPC3 CAR construct. (B–D) GPC3 CAR transduction efficiency in T cells was assessed by flow cytometry (B). To assess CAR‐T‐cell proliferation, cells were labeled with carboxyfluorescein succinimidyl ester (CFSE) and analyzed by flow cytometry. CAR‐T‐cell proliferation following treatment with or without PPG (200 μM, 24 h) was evaluated (C). The cytotoxicity of CAR‐T cells against HuH7 cells at the indicated effector‐to‐target (E:T) ratios, with or without PPG treatment (200 μM, 24 h), was showed in (D). All data were obtained from three independent biological experiments (*n* = 3). Statistical significance in (D) was analyzed using one‐way ANOVA followed by Tukey’s post hoc test. Data are presented as mean ± SD; error bars represent SD. *p* values are shown in the figure. (E–I) A schematic diagram of the in vivo experimental design for evaluating the effects of PPG on GPC3 CAR‐T‐cell activity against HuH7 xenografts is shown in (E). The tumor‐killing capacity of CAR‐T cells, with or without PPG treatment, was evaluated in a HuH7 xenograft mouse model (*n* = 6 per group). Representative images of excised tumors, tumor weights and tumor volume measurements are shown in (F–H). (I) Representative H&E and Ki67 stained tumor sections. Scale bars, 100 μm. Statistical significance for subfigures (G) and (H) was analyzed using one‐way ANOVA followed by Dunnett’s post hoc test, with each treatment group compared with the control group. Data are presented as mean ± SD; error bars represent SD. *p* values are shown in the figure;  ^∗∗∗∗^ *p* < 0.0001.

**Figure:**
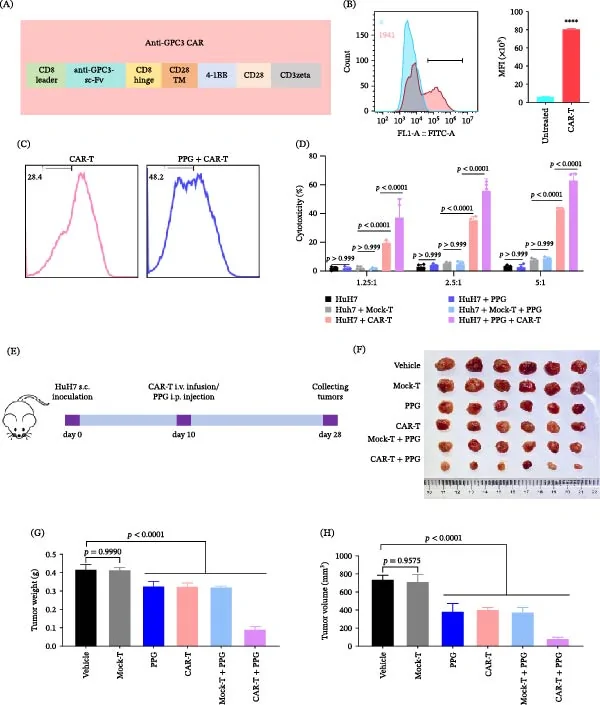

**Figure:**
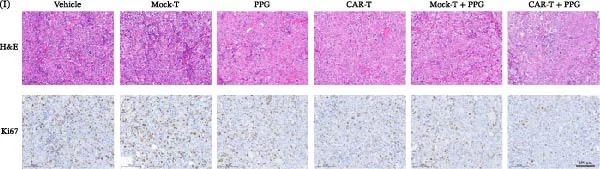

We further evaluated the antitumor effect of the PPG and CAR‐T combination in vivo. HuH7 cells were subcutaneously injected into immunodeficient mice, followed by the intravenous injection of CAR‐T cells on day 10 and the intraperitoneal administration of PPG. Tumor growth was monitored for 28 days. Compared with the vehicle group, the CAR‐T + PPG group showed greater antitumor activity than either PPG or GPC3 CAR‐T‐cell monotherapy (Figure 6E–H). Histological analysis by H&E staining revealed high tumor cell density and a compact tissue architecture in the vehicle and mock‐T groups (Figure 6I). In contrast, tumor tissues from the PPG, CAR‐T, and mock‐T + PPG groups exhibited reduced tumor cell density and a relatively looser tissue architecture (Figure 6I). Consistently, compared with the vehicle group, the mock‐T group showed no significant difference in the number of Ki67‐positive cells. In comparison with the vehicle group, Ki67 positivity was reduced in the PPG, CAR‐T, mock‐T + PPG, and CAR‐T + PPG groups, with the lowest level observed in the CAR‐T + PPG group (Figure S4).

To explore whether the antitumor effect of the PPG plus GPC3 CAR‐T‐cell combination exceeded that expected from independent treatment effects, we performed an exploratory Bliss independence analysis based on the endpoint tumor volumes. The expected tumor growth inhibition for the combination was 72.0%, whereas the observed inhibition was 89.2%, corresponding to a Bliss excess inhibition of 17.2 percentage points. The observed‐to‐expected residual tumor volume ratio was 0.385 (Table S5), indicating a lower residual tumor burden than that predicted under the Bliss independence model. Mock‐T and mock‐T + PPG groups were included as biological specificity controls and were not included in the Bliss analysis, which was restricted to the vehicle, PPG, CAR‐T, and CAR‐T + PPG groups. Exploratory Bliss analysis showed that the residual tumor burden in the combination group was lower than that predicted by the model; however, this finding does not establish pharmacological synergy. Collectively, these findings indicate that, in the HuH7 subcutaneous HCC xenograft model, the combination of PPG and GPC3 CAR‐T cells demonstrated greater antitumor activity than either PPG or GPC3 CAR‐T cell treatment alone.

As an effector cell in the antitumor immune response, CD8^+^ T cells have attracted increasing research attention regarding their role in the development and progression of liver cancer. We isolated human T cells and stimulated them with anti‐CD3 and anti‐CD28 for 48 h, followed by 24 h of drug treatment. We then examined the effect of PPG on exhaustion markers (PD‐1 and LAG‐3) and functional effector markers (TNF‐α and GZMB) in CD8^+^ T cells by flow cytometry. The gating strategy is shown in Figure S5. The results revealed that PPG treatment reduced the proportions of CD8^+^PD‐1^+^ and CD8^+^LAG‐3^+^ cells (Figure 7A–C) while increasing the proportions of CD8^+^TNF‐α^+^ and CD8^+^GZMB^+^ cells (Figure 7D–F). These results suggest that PPG may shift CD8^+^ T‐cell phenotypic markers toward a less exhausted and more effector‐associated profile.

**Figure 7:**
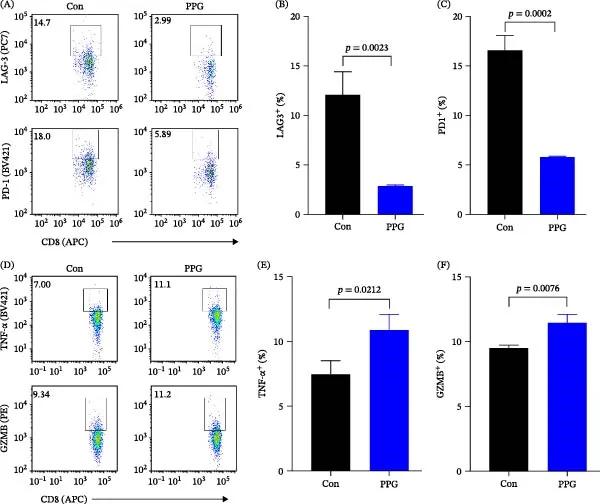
PPG affects CD8^+^ T cell exhaustion and effector‐associated phenotypes. (A–C) CD8^+^ T cells were treated with PPG (200 μM, 24 h), and the expression of the exhaustion markers LAG‐3 and PD‐1 was analyzed by flow cytometry. Representative flow cytometric plots are shown in (A), with quantification of CD8^+^LAG‐3^+^ cells and CD8^+^PD‐1^+^ cells shown in (B) and (C), respectively. (D–F) The effects of PPG treatment (200 μM, 24 h) on the effector markers TNF‐α and GZMB in CD8^+^ T cells were evaluated by flow cytometry. Representative flow cytometric plots are shown in (D), with quantification of CD8^+^TNF‐α^+^ cells and CD8^+^GZMB^+^ cells shown in (E) and (F), respectively. All data were obtained from three independent biological experiments (*n* = 3). For subfigures (B), (C), (E), and (F), statistical significance was analyzed using an unpaired two‐tailed Student’s *t*‐test, comparing the PPG‐treated group with the Con control group (equal volume of ddH_2_O). Data are presented as mean ± SD; error bars represent SD. *p* values are shown in the figure.

## 4. Discussion

H_2_S appears to exert concentration‐dependent effects on HCC. Previous studies have shown that low concentrations of H_2_S promote the migration of liver cancer cells, whereas high concentrations may be inhibitory [22]. Consistent with this observation, treatment with the slow‐releasing H_2_S donor GYY4137 (200 µM) enhanced the migration and invasion capabilities of HepG2 and HuH7 cells (Figure 1A). Overexpression of CBS, CTH, and MPST increased intracellular H_2_S levels, with the greatest increase observed in the CTH‐overexpressing cells (Figure 1K). These findings suggest that the pro‐migratory effect of CTH in HuH7 cells may be associated with the extent of H_2_S elevation. Additionally, the expression patterns of these enzymes have been reported to differ: CTH is mainly expressed in multiple organs, including the liver, kidneys, and cardiovascular system; the relative contributions of CBS, CTH, and MPST to H_2_S production are tissue‐, cell type‐, and context‐dependent [23]. Collectively, these findings suggest that the role of H_2_S is complex.

Rab7a is a small GTPase involved in multiple cellular processes, including cell growth, differentiation, autophagy, and apoptosis [24–26]. Numerous studies have demonstrated that this gene may serve as a potential risk biomarker for HCC [14, 27]. Furthermore, Rab7a exerts essential regulatory functions in critical signaling pathways, including EGF/EGFR [28, 29]. Our findings suggest that CTH/H_2_S signaling is associated with Rab7a sulfhydration. Using a previously established Rab7a triple mutant (CCC), in which Cys83, Cys84, and Cys143 were simultaneously replaced with serine, we found that CCC exhibited reduced Rab7a sulfhydration in HepG2 cells (Figure S2E), consistent with previous findings in HepG2.2.15 cells. These results suggest that Cys83, Cys84, and Cys143 may contribute to Rab7a sulfhydration. Notably, CTH did not further enhance cell migration in Rab7a‐overexpressing cells, possibly because Rab7a sulfhydration or its downstream signaling had reached a plateau under the experimental conditions. In contrast, CTH enhanced migration in CCC‐expressing cells, which may reflect the sulfhydration of endogenous Rab7a or the involvement of other H_2_S‐responsive targets. These possibilities require further investigation. We acknowledge that direct high‐resolution structural characterization, such as circular dichroism or nuclear magnetic resonance spectroscopy, was not performed to determine whether the Cys83/Cys84/Cys143 mutations induce local or global conformational changes in Rab7a.

Autophagy plays a complex and context‐dependent role in HCC, acting as both a tumor‐suppressive mechanism and a survival pathway that promotes cancer cell adaptation to metabolic stress [30]. In the present study, we observed that CTH overexpression altered LC3B and p62 levels as well as tandem mRFP‐GFP‐LC3 signals. However, these results are insufficient to fully distinguish reduced autophagosome formation from altered lysosomal degradation. Nevertheless, the precise stage of autophagy affected by CTH requires further clarification through complementary analyses of early autophagy signaling and lysosomal function. Future studies assessing autophagy initiation‐related signaling, together with lysosomal acidification, cathepsin activity, and LC3–LAMP1 colocalization, may help determine whether CTH affects late‐stage autophagic degradation.

CAR‐T cell therapy has emerged as a promising immunotherapeutic strategy for cancer [31]. Several studies targeting HCC with CAR‐T cells have demonstrated encouraging therapeutic potential [10, 11, 32]. GPC3 is highly expressed in HCC but has limited expression in normal human tissues [33]. Early‐phase clinical trial of GPC3 CAR‐T cells have reported acceptable safety profiles and preliminary antitumor activity in advanced HCC [34]. Combination strategies, including sorafenib plus GPC3‐directed CAR‐T therapy, have also shown potential clinical activity [35]. In our study, the combination of the CTH inhibitor PPG and GPC3 CAR‐T cells demonstrated greater antitumor activity than either monotherapy in the HuH7 subcutaneous xenograft model. Given the potential roles of the CTH‐H_2_S axis in tumor metabolism and immune regulation, subsequent studies will systematically evaluate the combination of PPG with anti‐PD‐1 antibodies and other metabolic modulators in xenograft models.

Key limitations affecting the translational interpretation of our findings include the lack of external validation of the observed associations between CTH expression and clinical outcomes as well as the unresolved mechanism of PPG‐mediated T‐cell modulation. First, the associations of CTH expression with clinicopathological features and patient survival were observed only in univariate analyses of a single‐center cohort; because external validation is lacking and center‐specific patient characteristics or treatment practices may have influenced these observations, the findings should be interpreted with caution. Future prospective studies incorporating larger multicenter cohorts with independent validation sets are warranted to confirm whether CTH provides independent prognostic information in HCC. Second, the mechanism by which PPG modulates T‐cell function remains incompletely understood. Although PPG reduced the expression of exhaustion‐associated markers and enhanced effector‐associated phenotypes in CD8^+^ T cells, the molecular basis of these effects requires further clarification. The current data do not determine whether these effects are mediated by inhibition of CTH within T cells or involve potential off‐target pharmacological effects and associated downstream signaling changes. Future studies using CTH loss‐of‐function and rescue experiments in primary T cells, together with tumor cell–T‐cell coculture systems and in vivo models, may help clarify the CTH dependency of PPG‐mediated T‐cell modulation, potential off‐target effects, and contributions from the tumor microenvironment. Metabolomic and transcriptomic analyses may also provide further insight into the underlying molecular mechanisms. Such studies may help define the biological contexts in which PPG could be used in combination with CAR‐T therapy and inform its translational interpretation.

## 5. Conclusions

Our findings support a role for CTH in liver tumor progression. In liver tumor cell models, CTH modulation was associated with changes in detected Rab7a sulfhydration levels and autophagy‐related markers. In the HuH7 subcutaneous xenograft model, combined treatment with PPG and GPC3 CAR‐T cells demonstrated greater antitumor activity than either monotherapy.

## Author Contributions

Xiaopan Wu and Ying Liu conceived the study. Yuxin Guo and Man Yu performed experiments for this study. Xilin Zhu and Wei Li performed staining of human samples and statistical analyses.

## Funding

This work was supported by grants from the CAMS Innovation Fund for Medical Sciences of China (Grants 2021‐I2M‐1‐016, 2018‐I2M‐1‐004, and 2016‐I2M‐1‐004) and the National Key Research and Development Program of China (Grant 2021YFC2700601).

## Disclosure

All authors read and approved the final manuscript.

## Conflicts of Interest

The authors declare no conflicts of interest.

## Supporting Information

Additional supporting information can be found online in the Supporting Information section.

## Supporting information


**Supporting Information** Figure S1: CTH promotes migration and invasion of HepG2 cells. This figure provides supporting data for the functional role of CTH. It demonstrates the effects of CTH overexpression, knockdown, and pharmacological inhibition (with PPG) on the migration and invasion capabilities of HepG2 liver cancer cells. Figure S2: Effects of Rab7a and CCC on HepG2 cells. The image illustrates the role of Rab7a and CCC in HepG2 cells. Figure S3: Effect of CTH on autophagy. Expression levels of LC3B in HepG2 and HuH7 cells following CTH overexpression, with or without bafilomycin A1 treatment. Figure S4: Quantification of Ki67‐positive cells in the subcutaneous tumor xenografts shown in Figure 6. Figure S5: Flow cytometry gating strategy. This figure illustrates the detailed gating strategy used for the flow cytometry analyses and for CD8 exhausted cells and effector cells. Table S1: List of antibodies. This table provides a complete list of all antibodies used in the study, including their specific targets, manufacturers, and catalog numbers. Table S2: List of flow cytometry antibodies. This table provides antibodies used in the flow cytometry of this study. Table S3: The compensation parameters of CD8 exhausted. Table S4: The compensation parameters of CD8 effector. Table S5: Exploratory Bliss independence analysis of endpoint tumor volumes for the PPG and GPC3 CAR‐T‐cell combination.

## Data Availability

All data generated or analyzed during this study are included in this published article and its supporting information files.
