# Supplementary material for: CTH Promotes Liver Cancer Progression and Exploratory Evaluation Supports Enhanced Antitumor Activity of PPG Plus GPC3 CAR‐T Cells
Source: J Immunol Res. 2026 Oct 16;2026:3351382. doi: 10.1155/jimr/3351382 (PMC13673299; doi:10.1155/jimr/3351382)
Supplement: Supplementary file 1 [file JIMR-2026-3351382-s001.docx]

**Supplement Materials**

Fig. S1: CTH promotes migration and invasion of HepG2 cells.

(A–C) CTH overexpression and knockdown in HuH7 cells (A) and HepG2 cells (B) were validated by Western Blot (WB). CTH expression in stably transfected HuH7 cells used for in vivo experiments was also determined by WB (C). WB was performed in three independent biological experiments (n = 3).

(D–F) The migratory capacity of HepG2 cells following CTH overexpression (D), CTH knockdown (E), or PPG treatment (200 μM, 24 h; F) was assessed using transwell migration assays. (G–I) The invasive capacity of HepG2 cells following CTH overexpression (G), CTH knockdown (H), or PPG treatment (200 μM, 24 h) (I) was assessed using transwell invasion assays. Migration and invasion assays were performed in three independent biological experiments (n = 3), and representative images from one experiment are shown. Scale bars, 500 μm. Representative images and the corresponding quantification of migrated or invaded cells are shown. For panels (D–I), statistical significance was analyzed using an unpaired two-tailed Student’s t-test, comparing each treatment group with its respective control group. Data are presented as mean ± SD; error bars represent SD. P values are indicated in the figure.

Fig. S2: Effects of Rab7a and CCC on HepG2 cells.

(A–B) HepG2 cells transfected with CTH or the corresponding control vector (A), or sh-CTH or the corresponding control vector (B), were treated with the H_2_S donor GYY4137 (200 μM, 24 h). Rab7a sulfhydration was subsequently assessed. WB was performed in three independent biological experiments (n = 3).

(C) Rab7a sulfhydration was assessed in HepG2 cells following Rab7a overexpression and CCC treatment. WB was performed in three independent biological experiments (n = 3).

(D–E) The migratory capacity of HepG2 cells following Rab7a overexpression (D) or CCC treatment (E) was assessed using transwell migration assays. (F–G) The invasive capacity of HepG2 cells following Rab7a overexpression (F) or CCC treatment (G) was assessed using transwell invasion assays. Migration and invasion assays were performed in three independent biological experiments (n = 3), and representative images from one experiment are shown. Scale bars, 500 μm. For all indicated two-group comparisons, statistical significance was analyzed using an unpaired two-tailed Student’s t-test. Data are presented as mean ± SD; P values are indicated in the figure.

Fig. S3: CTH overexpression affects LC3B expression in HepG2 and HuH7 cells.

LC3B expression was assessed by WB in HepG2 cells (A) and HuH7 cells (B) following CTH overexpression, with or without treatment with Bafilomycin A1 (Baf A1; 50 nM, 6 h). WB was performed in three independent biological experiments (n = 3), and representative blots from one experiment are shown.

Fig. S4: Quantification of Ki67-positive cells in subcutaneous tumor xenografts.

Quantification of Ki67-positive cells in subcutaneous tumor tissues from nude mice. The corresponding representative Ki67 immunohistochemical staining images are shown in Fig. 6. Tumor tissues from six mice per group were analyzed (n = 6), with one tumor section assessed for each mouse. Data are presented as mean ± SD. Statistical analysis was performed using one-way ANOVA followed by Dunnett’s post-hoc test, with comparisons made versus the indicated control group. P values are shown in the figure.

Fig. S5: Flow cytometry gating strategy and compensation parameters for CD8 exhausted cell and effector cell.

Briefly, doublets were first excluded using FSC-H/FSC-A and SSC-H/SSC-A parameters. Subsequently, immune cells were gated as PerCP/Cyanine5.5⁺ (CD45⁺) cells. From this population, CD8⁺ T cells were identified as FITC⁺ APC⁺ (CD3⁺ CD8⁺) cells. Finally, CD8⁺ T cell subsets were further characterized based on the expression of activation/exhaustion markers: APC/PE-Cy7⁺ (CD8⁺LAG-3⁺), APC/BV421⁺ (CD8⁺PD-1⁺ or CD8⁺TNF-α⁺) and APC/PE⁺ (CD8⁺GZMB⁺) populations.

**Table S1: List of antibodies.**

| **Antibody Name** | **Manufacturer** | **Catalog** |
| --- | --- | --- |
| GAPDH | Proteintech | 60004-1-1g |
| RAB7 | Proteintech | 55469-1-AP |
| LC3B | Abmart | T55992S |
| P62 | ABclonal | A19700-50-μL |
| Ki67 | Servicebio | GB121141-50 |
| CTH | Santa | Sc-374249 |
| CBS | Selleck | F1123 |
| MPST | UpingBio | YP-mAb-18581 |

The table lists the primary antibodies used in this study, including the target antigen, manufacturer, and catalog number.

**Table S2: List of flow cytometry antibodies.**

| **Antibody Name** | **Manufacturer** | **Catalog** |
| --- | --- | --- |
| PerCP/Cyanine5.5 anti-human CD45 Antibody | BioLegend | 368503 |
| FITC anti-human CD3 Antibody | BioLegend | 300406 |
| APC anti-human CD8a Antibody | BioLegend | 300911 |
| PE/Cyanine7 anti-human CD223 (LAG-3) Antibody | Biolegend | 369309 |
| [Brilliant Violet 421™ anti-human CD279 (PD-1) Antibody](https://www.biolegend.com/en-us/products/brilliant-violet-421-anti-human-cd279-pd-1-antibody-7191) | BioLegend | 367421 |
| PE anti-human/mouse Granzyme B Recombinant Antibody | BioLegend | 372207 |
| Brilliant Violet 421™ anti-human TNF-α Antibody | BioLegend | 502932 |
| anti-CD3 antibody | Biogems | 05121-25-500 |
| anti-CD28 antibody | Biogemes | 10311-25-500 |

The table lists the fluorochrome-conjugated antibodies used for flow cytometric staining of T-cell subsets and functional markers, as well as the anti-CD3 and anti-CD28 antibodies used for T-cell activation.

**Table S3: Compensation matrix for exhausted T cell markers.**

|  | **FITC-A** | **PC5.5A** | **PC7-A** | **APC-A** | **PB450-A** |
| --- | --- | --- | --- | --- | --- |
| **FITC-A** | 100 | 4.7569 | 0.6503 | 0.2006 | 0.1181 |
| **PC5.5A** | 0.2871 | 100 | 28.9207 | 2.2741 | 0.0645 |
| **PC7-A** | 0.8245 | 1.7949 | 100 | 0.3579 | 0.2281 |
| **APC-A** | 0.2257 | 3.0452 | 0.4801 | 100 | 0.656 |
| **PB450-A** | 0.074 | 0.043 | 0 | 0 | 100 |

Compensation was established using single-stained controls for the fluorochromes included in the T-cell exhaustion-marker panel. Values represent the compensation coefficients applied during flow cytometric data analysis. FITC, fluorescein isothiocyanate; PC5.5, PerCP/Cyanine5.5; PC7, PE/Cyanine7; APC, allophycocyanin; PB450, Pacific Blue 450.

**Table S4: Compensation matrix for effector T cell markers.**

|  | **FITC-A** | **PE-A** | **PC5.5-A** | **APC-A** | **PB450-A** |
| --- | --- | --- | --- | --- | --- |
| **FITC-A** | 100 | 31.5122 | 4.7569 | 0.2006 | 0.1181 |
| **PE-A** | 0.6489 | 100 | 17.9151 | 0.0107 | 0 |
| **PC5.5-A** | 0.2871 | 0.4121 | 100 | 2.2741 | 0.0645 |
| **APC-A** | 0.2257 | 0.3249 | 3.0452 | 100 | 0.0656 |
| **PB450-A** | 0.074 | 0.0574 | 0.043 | 0 | 100 |

Compensation was established using single-stained controls for the fluorochromes included in the T-cell effector-marker panel. Values represent the compensation coefficients applied during flow cytometric data analysis.

**Table S5: Exploratory Bliss independence analysis of endpoint tumor volumes for the PPG and GPC3 CAR-T-cell combination.**

| **Group** | **Mean Tumor Volume (mm³, mean ± SD)** | **Inhibition Rate (IR, %)** | **Expected IR (%)** | **Observed-to-expected residual tumor-volume ratio** |
| --- | --- | --- | --- | --- |
| **Vehicle** | 733.2 ± 53.0 | — | — | — |
| **PPG** | 377.7 ± 95.1 | 48.5 | — | — |
| **CAR-T** | 398.9 ± 28.4 | 45.6 | — | — |
| **CAR-T + PPG** | 79.1 ± 18.6 | 89.2 | 72 | 0.385 |

Tumor volumes are presented as mean ± SD (n = 6 mice per group). Tumor-growth inhibition and Bliss independence model–based expected inhibition were calculated relative to the Vehicle group as described in the Methods. Expected inhibition and the observed-to-expected residual tumor-volume ratio are shown for the combination group only. Mock-T and Mock-T+PPG groups were included as biological specificity controls and were not included in the Bliss analysis.
